# A Pap-Fed People

**Published:** 1901-08-24

**Authors:** 


### A PAP-FED PEOPLE.

One of the most interesting of the various papers read at the Cheltenham meeting of the British Medical Association was that by Dr. E. H. Van Someren, of Venice, in which, while preaching the old doctrine of slow eating and very thorough mastication, he gave certain reasons for his faith which imparted novelty and interest to his remarks. Turning to one of the most popular handbooks of physiology, that by Starling, we find the process of deglutition thus described: "When the food is sufficiently subdivided it is gathered by the movements of the tongue against the hard palate into a bolus which rests on the dorsal surface of the tongue, whence it is propelled through the fauces into the œsophagus;" and this description is evidently an accurate description of what usually happens. Mastication is thus regarded as a means of reducing the food to a condition fit for the action of the various digestive juices, and when the mouthful has been sufficiently chopped up and sufficiently moistened with saliva to enable it to go down, it is swallowed as a "bolus" at one gulp. According to Dr. Someren, however, this hardly represents what ought to happen. When mastication is properly performed, he says, the food passes to the back of the mouth and collects in the glosso-epiglottidean folds, where it remains in contact with the mucous membrane containing the sensory end-organs of taste, when such of it as has been properly reduced by the saliva is allowed to pass the fauces and is swallowed by a truly involuntary act of deglutition, while such as has not been sufficiently insalivated ought to be returned into the mouth again for the process to be repeated. He says that this should occur again and again in the course of the mastication of each mouthful, until all sense of taste has been removed from it, and then only should the remaining mass of fibre or husk or connective tissue be swallowed by a conscious effort. By the constant habit of bolting our food we have to a large degree lost this reflex power, which ought to be possessed by the fauces, of sorting out the products of mastication and refusing to pass such food as still retains its sapidity, and as a result we suffer from indigestion and all sorts of evils besides making our internals a reservoir of decomposing food from which we obtain no nutriment whatever. If then we would be rid of these evils, which extend far beyond the more immediate consequences of indigestion, we must chew our food carefully and well, not merely giving the thirty-two bites a mouthful to which according to tradition the long life and prolonged vigour of the late Mr. Gladstone was largely due, but steadily continuing the act of mastication until every particle of taste has been removed from the food. Then and then only may the residue be swallowed. Dr. Someren assures us that although at first this may be a wearisome affair, the instinct or the reflex will before long be re-developed which we ought never to have lost, by aid of which the whole thing will become automatic, the fauces involuntarily returning into the mouth all that is not in a fit state for swallowing. He prophecies that dyspepsia and all its consequences will cease to exist if patients be persuaded to bite their food until its original taste disappears or is carried away by involuntary deglutition. But ought we not to train our children from the beginning in this art? In the first few months of life, when saliva is not secreted, Nature ordains that the infant shall be fed on the mammary secretion which is alkaline. But with the eruption of teeth and the occurrence of an abundant flow of saliva there comes a capacity for dealing with other kinds of food, and if people did not interfere and insist on baby only taking sloppy, pappy kinds of food, the infant would share its mother's meals, would exercise its masticatory muscles upon tough crusts, and even gnaw the bones off its mother's plate, spending hours probably in the process, hours of enjoyment and of development. But, no! The dictum has gone forth that the proper food for babes is pap, that whatever it has must be soft and easy to swallow. Thus we rear a pap-fed people, trained from their earliest days to bolt their food, and fated all their lives to suffer from indigestion.

